# Strength Restoration of Cracked Sandstone and Coal under a Uniaxial Compression Test and Correlated Damage Source Location Based on Acoustic Emissions

**DOI:** 10.1371/journal.pone.0145757

**Published:** 2015-12-29

**Authors:** Xiaowei Feng, Nong Zhang, Xigui Zheng, Dongjiang Pan

**Affiliations:** School of Mines, Key Laboratory of Deep Coal Resource Mining, Ministry of Education, China University of Mining and Technology, Xuzhou, China; China University of Mining and Technology, CHINA

## Abstract

Underground rock masses have shown a general trend of natural balance over billions of years of ground movement. Nonetheless, man-made underground constructions disturb this balance and cause rock stability failure. Fractured rock masses are frequently encountered in underground constructions, and this study aims to restore the strength of rock masses that have experienced considerable fracturing under uniaxial compression. Coal and sandstone from a deep-buried coal mine were chosen as experimental subjects; they were crushed by uniaxial compression and then carefully restored by a chemical adhesive called MEYCO 364 with an innovative self-made device. Finally, the restored specimens were crushed once again by uniaxial compression. Axial stress, axial strain, circumferential strain, and volumetric strain data for the entire process were fully captured and are discussed here. An acoustic emission (AE) testing system was adopted to cooperate with the uniaxial compression system to provide better definitions for crack closure thresholds, crack initiation thresholds, crack damage thresholds, and three-dimensional damage source locations in intact and restored specimens. Several remarkable findings were obtained. The restoration effects of coal are considerably better than those of sandstone because the strength recovery coefficient of the former is 1.20, whereas that of the latter is 0.33, which indicates that MEYCO 364 is particularly valid for fractured rocks whose initial intact peak stress is less than that of MEYCO 364. Secondary cracked traces of restored sandstone almost follow the cracked traces of the initial intact sandstone, and the final failure is mainly caused by decoupling between the adhesive and the rock mass. However, cracked traces of restored coal only partially follow the traces of intact coal, with the final failure of the restored coal being caused by both bonding interface decoupling and self-breakage in coal. Three-dimensional damage source locations manifest such that AE events are highly correlated with a strength recovery coefficient; the AE events show a decreasing tendency when the coefficient is larger than 1, and vice versa. This study provides a feasible scheme for the reinforcement of fractured rock masses in underground constructions and reveals an internal mechanism of the crushing process for restored rock masses, which has certain instructive significance.

## Introduction

Thanks to continuous sedimentation effects and tectonic movements that have occurred on the scale of billions of years, the Earth’s strata show a general trend of stability, even though seismic activities and volcanism are dispersed worldwide. However, artificial underground excavations within the earth significantly redistribute the strata’s stresses, and these excavations can vary according to their different construction purposes, such as radioactive waste disposal, underground hydraulic projects, tunnels, subways and shallow-burial underground facilities. In particular, underground excavations for coal resources considerably differ from those for other engineering applications. The stability of tunnels, subways and shallow-burial underground facilities should be sustained for multiple generations, and dynamic monitoring for more than a century for a nuclear waste repository is fairly normal [1–3]. In contrast, the service life of most roadways or entries in a coal mine is only a few years or even months (provided that excavations such as main haulage roadways or main return airways likely should have a service life of more than several decades) [4] because all excavations are constructed for coal hauling. Hence, a certain amount of deformation is permissible. In addition, leaving the free deformation of the surrounding rock alone for a certain amount of time after a specified space is shaped by an integrated excavation is recommended. The main explanation for this approach is the so-called pressure relief support methodology, which is frequently adopted for deep coal mines (with a general depth of more than 1,000 m) under high crustal stress [5]. The initially high pressure in the peripheral strata is released first; subsequently, the support scheme is used to restrain further deformation of the rock mass. The support structures generally comprise bolts, cables, U-shaped supports, steel nets, or different combinations.

**Fig 1:**
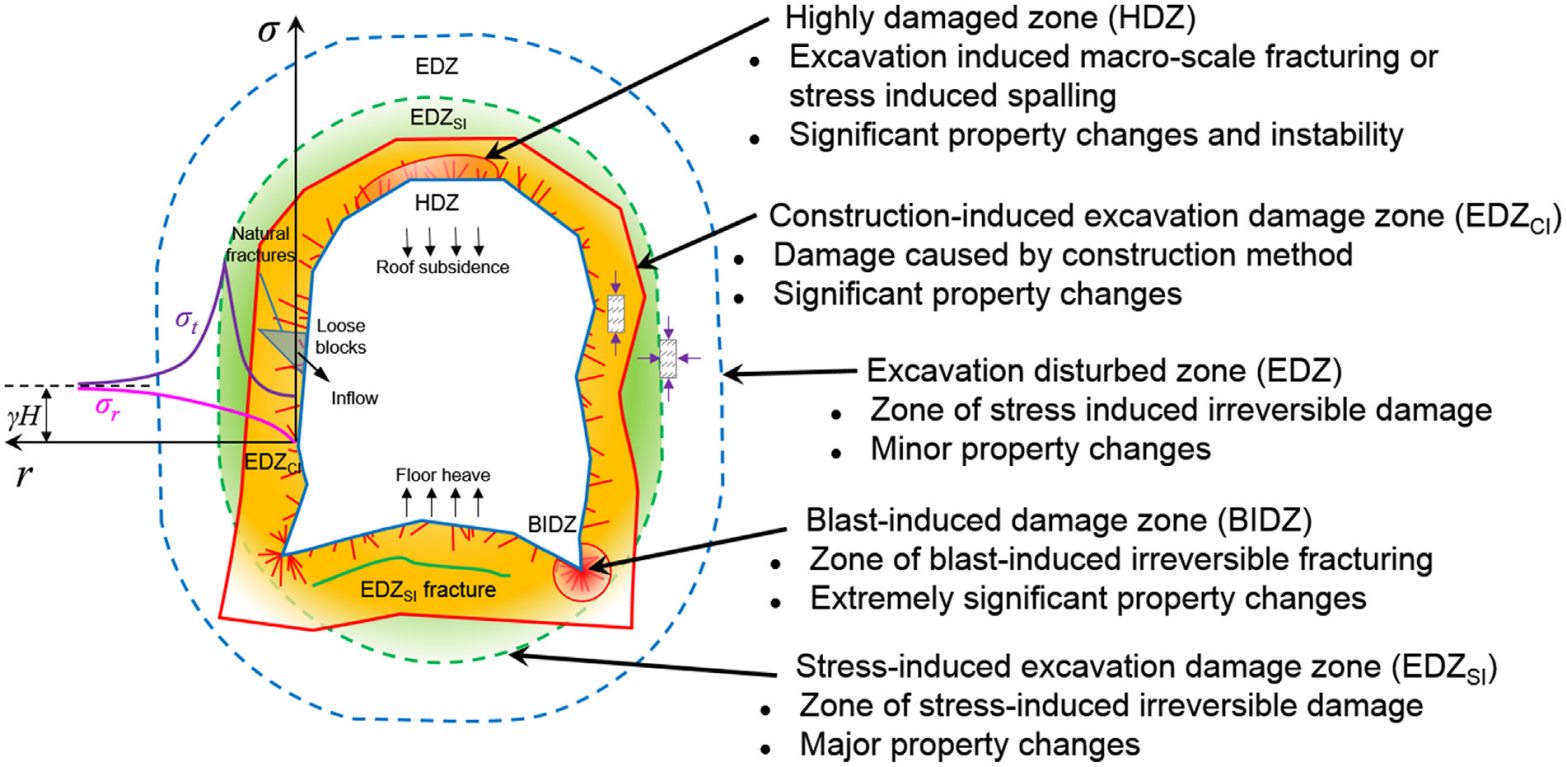
Overview of the different damage zones around an underground excavation, where *σ*_*r*_ is the radical stress, *σ*_*t*_ is the tangential stress, *γ* is the average bulk force of the overlying strata, and *H* is the burial depth of the excavation (adapted from the work by [9])

Rock is a heterogeneous material, and a large number of microscopic fissures and inherent pores are irregularly distributed in it. The three-dimensional stress in a natural rock is relatively balanced, and man-made excavation destroys this balance, turning it into two-dimensional stress or one-dimensional stress [6]. According to field observations, three basic types of failure modes exist: splitting, spalling, and oblique failure [7]. All these failures are a single form or combination of evolutionary processes that progress from microscopic cracks to macroscopic cracks [8]. The surrounding rock shows distinct districts of differentiation in terms of different degrees of breakage, as shown in Fig 1 [9]. Rock in the construction induced excavation damage zone (EDZ_CI_) is severely different from its original state; this zone is also called the breakage zone, where the property of the rock mass is irreversibly changed, and the media here show a general trend of instability. The stress that is applied to the breakage rock is general in its one- or two-dimensional stress state, and the stress strength is relatively low compared to its primary state. Considering a radical direction, the rock mass will sequentially express a plastic property, in which the stress environment of most of the rock mass belongs to a three-dimensional state, which is the stress-induced excavation damage zone (EDZ_SI_) in Fig 1. Macroscopic cracks rarely occur in this area, but microscopic cracks that are internal to the rock are adequately developed, and these inner cracks provide permeable channels for secondary restoration within the rock, such as superfine cement and chemical reinforcement agents [10]. This stress will increase as the distance to the edge of the excavation increases, and the peak value of the tangential stress is considerably higher than its initial value, which is mainly induced by stress redistribution. If we move further along in the radical direction, we next consider the elastic zone, or excavation disturbed zone (EDZ), in the figure; the rock property in this area is similar to the primary rock mass, and both the radical stress and tangential stress tend to retrieve their original rock stress, as illustrated by the curve in Fig 1. Overall, resisting man-made excavation and attempting to return to its initial intact balanced state is in the nature of the rock; thus, the occurrence of roof subsidence, floor heaving, and rib convergence in a coal mine roadway is normal [5].

Abandoning an underground space whose surrounding rock mass has been fully broken and then excavating another chamber/roadway/tunnel is unwise. The typical approach is to adapt some intensive support schemes; however, this approach still belongs to passive support, and fracture propagation in the rock mass cannot be restricted. Recently, grouting has been determined to be an effective method of restoring the peripheral fractured rock mass [11,12]. Grouted seriflux can fill cracks among granules [13], and the coalescence and propagation of microscopic cracks becomes limited. Then, these crushed rock masses can stick together as an intact mass with a certain bearing capacity to stabilize the normal cross-section of the underground space.

Recent studies have found that brittle materials, such as rock, glass, and similar substances, can release transient elastic waves when they experience an external force or internal force, which is mainly induced by elastic deformation and crack initiation/propagation in the material; this elastic wave emission is also called acoustic emission (AE) [14]. The waveform of an AE carries information regarding the location, growth distance, velocity and orientation of the cracks. Conventional descriptions of rock failures mainly focus on stress and strain measurements, which are not sufficiently precise. Hence, increasing numbers of researchers are attempting to evaluate rock failure with the aid of AE monitoring systems and to investigate crack initiation, propagation, and coalescence in rocks that are subjected to loading [15,16].

In this study, we investigate the engineering properties of intact sandstone and coal specimens under uniaxial loading; based on the initial results, these fractured specimens are restored by MEYCO 364 and then followed by a secondary uniaxial loading test. The strain-stress acquisition system and an AE detection system work together during this testing procedure. The strength recovery coefficient and the restoration effectiveness of coal and sandstone are discussed, and the inner fracturing mechanism and a damage source comparison before and after restoration are analyzed. The objectives of these experiments are to determine the restoration ability of adhesive on fractured rock masses and provide additional insight for practical engineering.

## Materials and Methods

### Specimen preparation and test measurements

**Fig 2:**
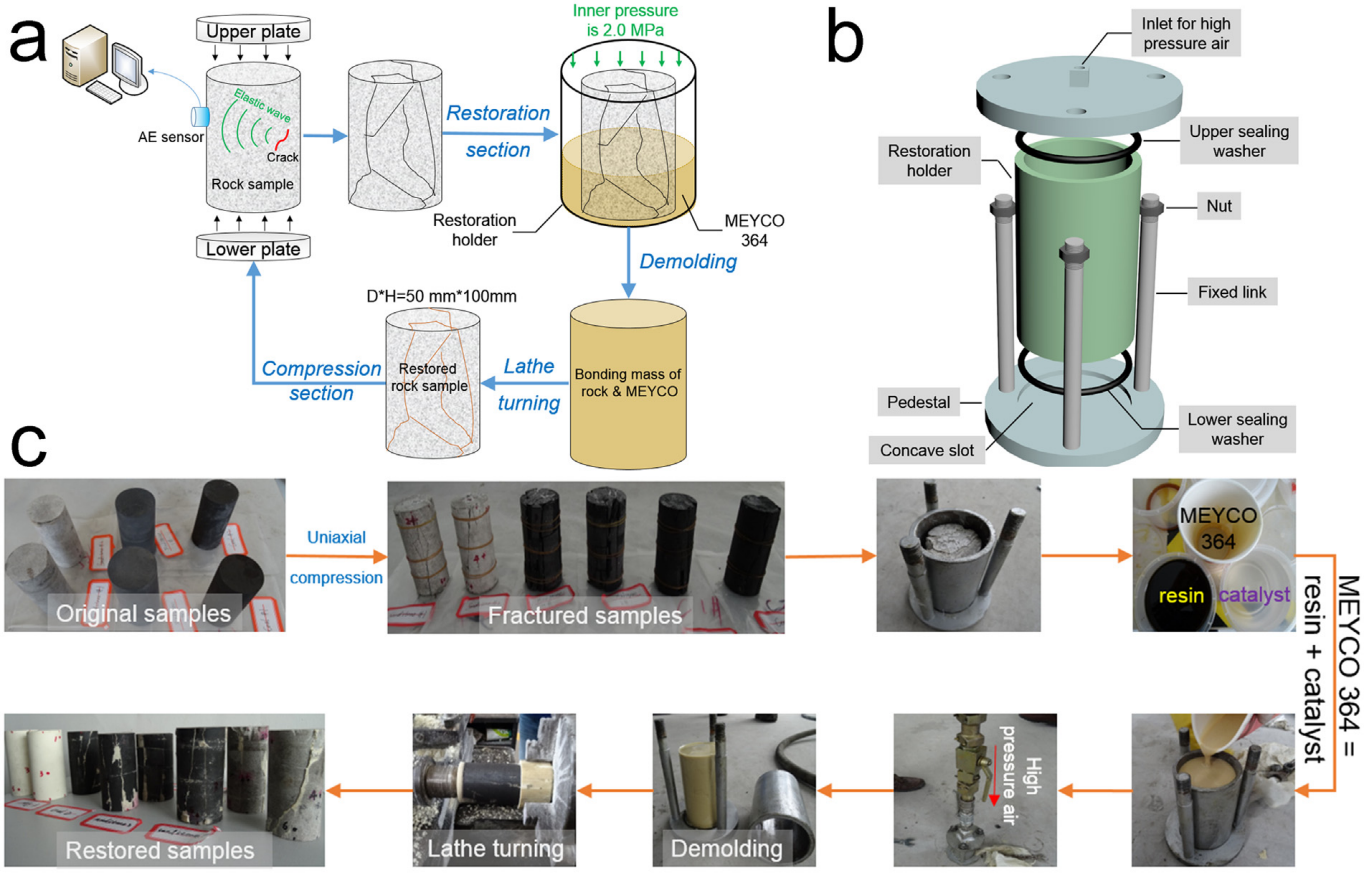
Schematic diagram of the technical process for the experiment. **(a)** Compression and restoration on the rock samples. **(b)** 3D view of a device for sample restoration, where the height, inner diameter, and outer diameter for the restoration holder are 110, 60, and 70 mm, respectively. The thickness and diameter of the pedestal are 10 and 110 mm, and the depth of the concave slot is 3 mm, which is consistent with the lower sealing washer. **(c)** Practical experimental routine for coal, sandstone, and mudstone. The volume ratio between the resin and catalyst is 1, the pressure of the high-pressure air is 2.0 MPa, and the inner wall of the restoration holder is evenly coated with Vaseline to facilitate the demolding process.

The specimens comprised three different groups: two cylindrical coal specimens, two cylindrical sandstone specimens, and two cylindrical mudstone specimens (the results for mudstone were inauthentic because of an air leakage problem during the restoration process on the cracked mudstone; hence, this study focuses on the sandstone and coal specimens). Each group was drilled from the same borehole around the shaft gate of the Kongzhuang Mine, Datun Coal Power Company, where the burial depth was at least 1,000 m. Some sites around the testing area showed a tendency to undergo rock burst. The specimens were prepared based on the ISRM-suggested method [17]; these specimens were right circular cylinders that had a height-to-diameter ratio of 2.0 and a diameter of 50 mm, with the ends of these specimens polished to be flat to ±0.01 mm. Some inevitable machining tolerances occurred because of processing imperfections; nevertheless, the tolerance could be neglected in view of its unapparent alteration of the final testing results. We chose a chemical high-polymer adhesive called MEYCO 364, which has widespread applications in geotechnical engineering areas worldwide [18], especially for rock or gravel reinforcement in coal mine roadways, tunnel concrete cracks, and underwater constructions, to present a secondary restoration on these specimens after the first comprehension test. The dilatation coefficient, cohesive strength, and compression strength of this adhesive exceed 1, 3.0 N/mm^2^ and 45 N/mm^2^, respectively. High-polymer material has many advantages compared to its alternatives, such as superfine cement, quick setting cement, and Portland cement [19–21] (which is mainly produced by the BASF HOCK Mining Chemical Company Ltd., China). We invented a device that is capable of injecting MEYCO 364 into the inner cracks of the specimens to restore the strength of the breakage specimens from uniaxial compression, a schematic illustration of which is shown in Fig 2(b). The flowing and coagulation times for MEYCO 364 are only 120±30 s and 150±30 s, respectively; hence, the restoration section should be rapidly processed to guarantee better liquidity and injection effects. The technical process for the experiment is illustrated in Fig 2, and the parameters of certain analyzed specimens are shown in Table 1.

**Table 1:** Physical and mechanical properties of the test samples.

Specimens	Categories	Diameter (mm)	Height (mm)	Cross-sectional area (mm^2^)	Weight (g)	Volume (mm^3^)	Density (kg∙m^-3^)
Coal No. 2	Intact	49.07	100.65	1891.13	255.10	1.91E5	1335.60
Coal No. 2	Restored	49.55	97.42	1928.31	245.05	1.88E5	1303.46
Sandstone No. 1	Intact	48.32	101.24	1833.77	465.70	1.86E5	2503.76
Sandstone No. 1	Restored	48.67	99.68	1867.31	465.60	1.86E5	2503.23
MEYCO 364	Intact	50.01	100.20	1964.28	258.90	1.97E5	1314.21

### Test equipment

**Fig 3:**
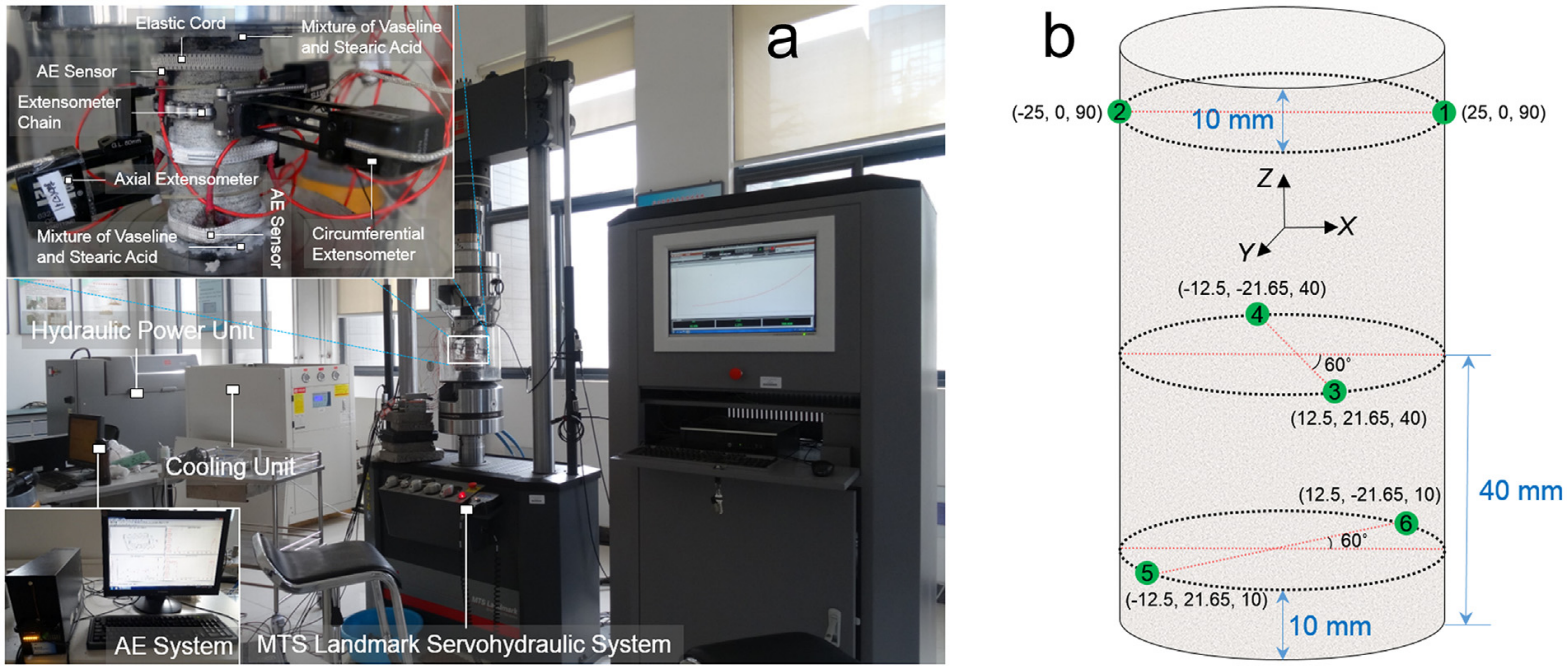
Layout of the testing apparatuses and AE sensors around the specimens. **(a)** Layout of the extensometers around the specimens (top left view), compression system, and AE system (bottom left view). **(b)** Location parameters for the acoustic sensors around a specimen, in which the green dots represent AE sensors. The sensors were fixed by elastic cords, as shown in the top left view in Fig 3(a).

Strain foils are typically adopted to measure the lateral strain and axial strain of a specimen; the limitations of this method are its low accuracy, tedious preliminary work, and high failure rates. Here, an MTS Landmark Servohydraulic System that was made by the MTS System Corporation (Minnesota, USA) was utilized, which we hoped would provide better results. This system was divided into the hydraulic power unit, cooling unit, and computing unit, as shown in Fig 3(a). The axial extensometer can measure the exact axial strain, whereas the circumferential extensometer’s capability is considerably different. This difference arises from the phenomenon that the circumferential extensometer measures the change in the chord length between the centers of the two end rollers in the chain when the specimen deforms radically under uniaxial compression; this value is not the change in the specimen’s circumference. The compensation calculation for this difference is described in the S1 File. In addition, two loading modes for the uniaxial compression test were present in this MTS machine, which are the load-controlled mode [22] and displacement-controlled mode [23]. The specimen might show a relatively short breakage duration under the load-controlled mode [24]. Thus, fewer AE events can be captured, so we adopted the displacement-controlled mode to stably proceed to the uniaxial compression. The rate was 0.1 mm/min.

The AE system was made by the American Physical Acoustic Corporation (MISTRAS Group Inc., Princeton Junction, USA), and the AE sensor was a Nano 30 sensor with a frequency domain of 125–750 kHz. The threshold, pre-amp analog filter, and sample rate were 40 dB, 100–400 kHz, and 0.5 MHz, respectively. The peak definition time (PDT), hit definition time (HDT), and hit lockout time (HLT) were set to 50, 200, and 300, respectively; the PDT, HDT and HLT are the timing parameters of the signal measurement process. A cylindrical specimen should have at least three sensors attached to locate the damage source. In this study, six sensors were used to optimize the AE source location’s accuracy; their spatial distribution around the specimen is shown in Fig 3(b). On a microscopic scale, the surfaces of the sensor and the specimen are quite rough, and only a few spots are actually in contact. The microscopic gaps were filled with a full layer of TM-100 ultrasonic coupling agent to improve their coupling effects. Additionally, AE events around the end areas can be detected in an early stage of uniaxial compression; this phenomenon will disturb the initial AE emission detection accuracy, and these end effects can significantly dominate the overall AE location pattern if a stress concentration occurs [25]. Several potential solutions can eliminate these end effects (noise from fretting between the upper compression plate and upper end of the specimen or between the lower compression plate and lower end of the specimen) as much as possible, such as attaching lead foil [26], Vaseline [27], grease, or rubber material [28] between them. We utilized a mixture of Vaseline and stearic acid to further improve the accuracy of the damage source location; we chose a volume ratio of 1:1 to better eliminate the end effects during the loading procedure.

## Results and Discussion

Different AE parameters can be used to evaluate the propagation from inherent microscopic fissures to macroscopic cracks in rock masses, such as the AE count, AE energy, amplitude, rise time, and duration time. These parameters have been documented in previous research studies. Rock masses “talk” when they experience stress, and humans can “listen” to the sounds of fissures growing with the aid of AE equipment. These crucial parameters enable the detection of small-scale damage long before a final failure.

Because AE energy is more sensitive to both the amplitude and the duration, it is simultaneously less dependent on the voltage threshold and operation frequencies. Hence, the AE energy is considerably better than the AE count for analyzing the magnitude of a source event. In this study, both the AE energy and accumulative energy were adopted to present a better explanation. The accumulative energy is the more convenient approach for evaluating the total emission quality, whereas the AE energy highlights the changes in the activity that occur during each moment of the test.

### Test results for sandstone specimens

Difficulty and deviations would arise if we relied solely on the stress-strain curve to manually identify transitional thresholds, such as crack closures or crack initiation thresholds. Nevertheless, the crack damage threshold can be identified based on the volumetric strain-stress curve [29]; the crack damage threshold is the moment at which critical energy is released and is followed by unstable fracture propagation [30]. At this moment, the volumetric strain curve will stop its negative extension and start a positive extension; a negative value means volume contraction, and a positive value means volume dilatation [31]. The crack damage threshold can also be defined as the reversal sign in an increment along the volumetric strain curve [32]. Historically, the volume change of a rock under compression was initially monitored by Bridgman, who conducted a uniaxial compression test on soapstone, marble, and basalt and ascribed this phenomenon to the structural changes that were internal to the rock. This change is also a preliminary sign of a macroscopic fracture [33].

#### Test on intact sandstone

**Fig 4:**
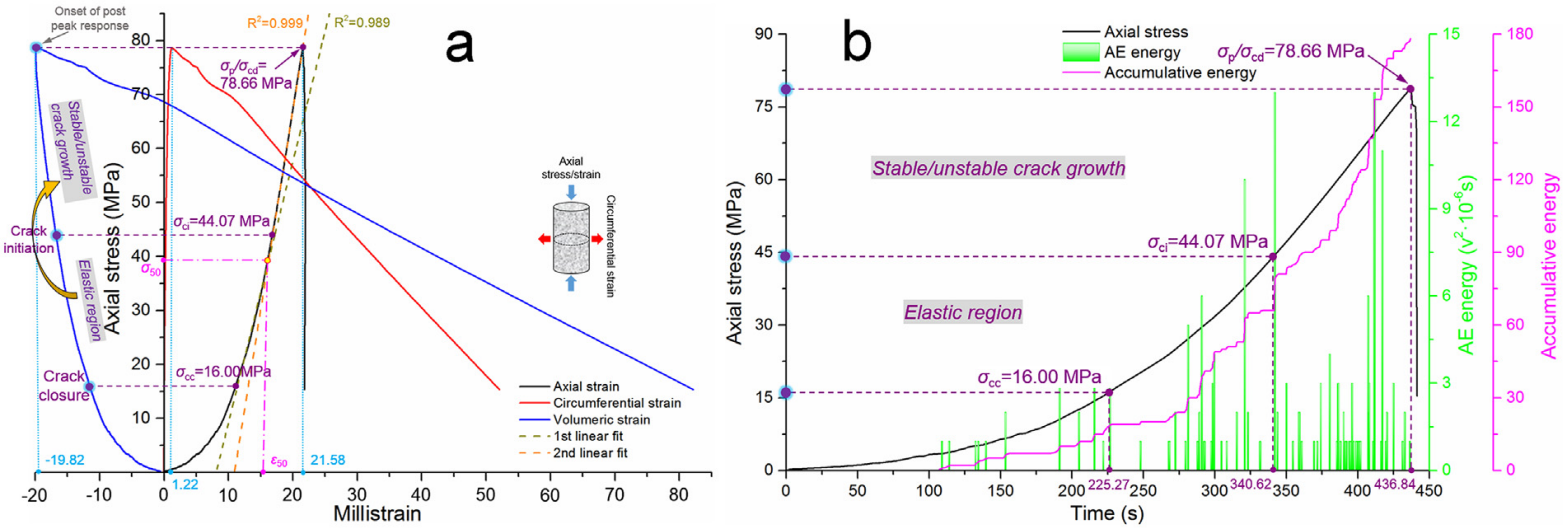
Test results of intact sandstone No. 1. **(a)** Relationship between the axial/circumferential/volumetric strain and axial stress under uniaxial compression. **(b)** Relationship between the time and axial stress/AE energy/accumulative energy under uniaxial compression.

Hoek and Bienawski [34] found that the crack propagation process included several stages, such as crack closure followed by an elastic region, crack initiation followed by stable crack propagation, crack damage followed by unstable crack propagation and ultimate failure. According to the test results on intact sandstone, we found certain similarities between sandstone No. 1 and sandstone No. 2, which can be ascribed to their identical drilling core; thus, they share similar mechanical properties and inherent crack morphologies. Fig 4 shows the curves of sandstone No. 1, as seen in Fig 4(a). The volumetric strain-stress curve shows no distinct reversal, which can mark a crack damage threshold (*σ*_cd_) and distinguish the peak stress (*σ*_p_). The reversal in the volumetric strain is located at the extreme point of the curve, where the horizontal tangent line of this point also meets the extreme points of the circumferential strain curve and axial strain curve. Because the sampling sites of these specimens were very deep (-1,015 m underneath the surface) and because the Kongzhuang Mine has some rock burst histories with different magnitudes, *σ*_cd_ and *σ*_p_ may occur simultaneously under uniaxial compression, which indicates that this type of sandstone has a certain tendency to produce rock bursts. The crack closure threshold (*σ*_cc_) and crack initiation threshold (*σ*_ci_) can be defined based on Fig 4(b), and a small amount of AE energy was detected in the time range of 0–100 s; this procedure accounts for 22.90% of the total pre-peak test time. According to the first non-linear section in the stress-strain curve in Fig 4(a), *σ*_cc_ can be defined as the point in the time-accumulative energy curve at which the accumulative energy reaches its first plateau, as labeled in Fig 4(b). In the same measurement units, *σ*_ci_ is defined when the time-accumulative energy curve transitions to a sharp increasing trend and after which no further intermediate plateau occurs. Based on the aforementioned analysis, the ratios for *σ*_cc_/*σ*_p_, *σ*_ci_/*σ*_p_, and *σ*_cd_/*σ*_p_ are 0.20, 0.56, and 1, respectively. The overall trend in the AE energy curve demonstrates that infrequent AE energy occurred before the crack closure threshold, and a large number of cracks would have propagated and coalesced with one another during the stable/unstable crack growth before the onset of the post-peak response; the AE energy during this stage was considerably greater than that during the elastic stage.

An elastic modulus, which is an essential factor in characterizing rock mechanics, is introduced to present a detailed evaluation of the mechanical properties of the specimens. Each elastic modulus is obtained based on the linear portion of the stress-strain curve. However, manually determining this portion may be difficult under certain circumstances, and the curve could show an overall non-linear and concave trend before it reaches its peak stress. Hence, two additional parameters are introduced. The first parameter is the average elastic modulus, which is the average slope that is calculated by the *σ*_cc_-*σ*_ci_ slope and *σ*_ci_-*σ*_p_ slope. The second parameter is the secant elastic modulus, which is the slope of the line that connects the origin and the point where the stress accounts for 50% of the peak stress. The equations for these parameters are expressed as follows:

$$\bar{E}=\frac{E_{1}+E_{2}}{2}$$ (1)

$$E_{50}=\frac{\sigma_{50}}{\varepsilon_{50}}$$ (2)

where$\bar{E}$, *E*_1_, and *E*_2_ are the average modulus, *σ*_cc_-*σ*_ci_ slope, and *σ*_ci_-*σ*_p_ slope, respectively. *E*_50_, *σ*_50_, and *ε*_50_ are the secant elastic modulus (MPa); axial stress, whose value accounts for half of the peak stress (MPa); and axial strain, whose corresponding axial stress is half of the peak stress, respectively.

The calculation results based on the aforementioned analysis are listed in Table 2.

**Table 2:** Key testing parameters for intact sandstone and restored sandstone (No. 1).

Specimen	*σ*_cc_ (MPa)	*σ*_ci_ (MPa)	*σ*_cd_ (MPa)	*σ*_p_ (MPa)	*E* (GPa)	*E*_50_ (GPa)	MV* (E-3)	MC* (E-3)
Intact sandstone	16.00 (0.20)^#^	44.07 (0.56)	78.66 (1.00)	78.66	6.12	2.44	-19.82	1.22
Restored sandstone	7.17 (0.27)	17.82 (0.68)	25.13 (0.96)	26.13	3.74	1.34	-11.95	1.35
Specimen	MA* (E-3)	TC* (s)	TI* (s)	TD* (s)	TP* (s)	TC-TI* (s)	TI-TD* (s)	TD-TP* (s)
Intact sandstone	21.58	225.27	340.62	436.84	436.84	115.35	96.22	0
Restored sandstone	13.16	155.84	213.32	244.79	259.83	57.48	31.47	15.04

^#^ Value in the bracket is defined as the ratio between the axial stress of each threshold and the peak stress.

* MV, MC, and MA represent the maximum absolute volumetric/circumferential/axial strain before fracturing, respectively. TC, TI, TD, and TP are the moments when *σ*_cc_, *σ*_ci_, *σ*_cd_, and *σ*_p_ are reached, respectively, whereas TC-TI, TI-TD, and TD-TP are the duration between TC and TI, TI and TD, and TD and TP, respectively.

#### Test on the restored sandstone

**Fig 5:**
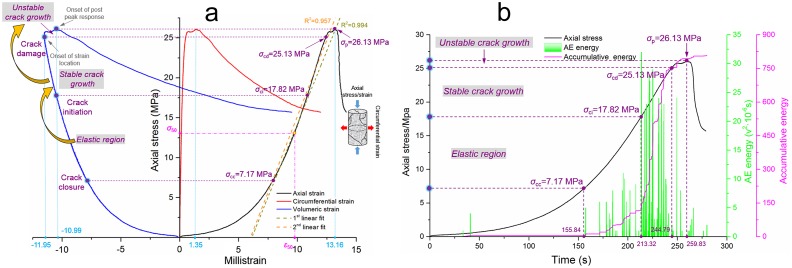
Test results for the restored sandstone No. 1. **(a)** Relationship between the axial/circumferential/volumetric strain and axial stress under uniaxial compression. **(b)** Relationship between the time and axial stress/AE energy/accumulative energy under uniaxial compression.

After the first round of testing on the initial intact sandstone No. 1, the results showed a fractured mode. This cracked sandstone was restored accordingly, and the relative operation process is represented in the ‘Specimen preparation and test measures’ section. The test results on the restored sandstone No. 1 are shown in Fig 5. As seen in Fig 5(a), clear differences with the results of the intact sandstone No. 1 arise because the crack damage threshold shows an outward trend and the strain-stress curve shows an overall concave trend. The occurrence of a crack damage threshold means the relief of the rock burst trend, whereas the overall concave trend indicates a large number of existing inner cracks. Correlated mechanical parameters are calculated by using Eqs (1) ~ (2) and are listed in Table 2.

Using the same method as described above, the crack closure threshold (*σ*_cc_) and crack initiation threshold (*σ*_ci_) are defined by a combination analysis on the strain-stress curve and AE time-accumulative curve. Whereas the crack damage threshold (*σ*_cd_) is defined by the first reversal sign along the volumetric strain-stress curve, stable crack propagation at this point will be replaced by unstable crack growth, and the turning point is indicated by the onset of the strain, as shown in the figure. The peak stress of the restored sandstone is 26.13 MPa, and the strength recovery coefficient (the ratio between the initial peak stress and restored peak stress) is 0.33, which indicates that the breakage strength in sandstone can be partially retrieved by a grouting measure; this approach has significance in providing guidance and meaning for practical engineering.

With regard to the volumetric strain-stress curve and circumferential curve, the maximum negative strains (volume dilatation induced) before and after restoration are -19.82E-3 and -11.95E-3, respectively, whereas the corresponding circumferential strains and axial strains are 1.22E-3 and 1.35E-3 for the former and 21.58E-3 and 13.16E-3 for the latter. Based on a comparison, these findings indicate that the decrease in volumetric strain manifests such that the dilatation effect is mitigated after restoration. However, the circumferential strain shows a slight increment along the horizontal direction; hence, the fracturing procedure for restored sandstone is mainly dominated by the propagation of vertical cracks. This phenomenon can be explained from two perspectives. The first explanation follows a penny-shaped crack version [35] or tensile failure [8]. The second explanation should be ascribed to the specific initial fracture morphology and bonding interface between the MEYCO-364 and sandstone, but this approach requires further discussion because of the decrease (39.02%) in the axial strains. This problem will be investigated further in the ‘Damage source location between the intact and restored sandstone’ section.

By comparing Figs 4(b) and 5(b), the most notable difference in the AE energy emission is the different distribution range and amplitude. The restored sandstone shows relatively minor AE activity during its crack closure course, and then the AE emission starts to increase and maintain this growth until its pre-peak stress, whereas the intact sandstone shows a uniform distribution throughout its uniaxial compression procedure. In addition, both the amplitude of the AE energy and the accumulative energy of the restored sandstone show an advantage compared to intact sandstone. An explanation for the aforementioned phenomenon is that the intact sandstone is not homogeneous and isotropic on a microscopic level, and different amounts of inherent cracks were generated during its millions or billions of years of evolution. Hence, some AE activities can be detected before the crack closure threshold, and it is normal for a natural intact rock mass to express uniform AE activity distribution until the final fracture [36–38]. However, a restored rock mass with some reinforcement materials, such as an MEYCO-364 injection, can also exhibit fracture sandstone changes in the distribution proportion of the inner cracks, and most of the macroscopic cracks and some microscopic cracks inside the sandstone are filled up with adhesive material. In addition, MEYCO 364 shows a clear plastic characteristic (see ‘Test results for MEYCO 364 specimens’), and the initial mechanical properties are dramatically altered by the blend of the plastic adhesive. Nevertheless, this injection cannot reach an absolute fill in many of the cracks because of various factors, such as inner air in the cracks, the injection pressure, and device leakproofness. Hence, some unfilled macroscopic cracks close during a relatively quiet period, and afterward, the crack closure turns into a closure initiation. The specific internal mechanism will be discussed in the next section.

The test results are summarized in Table 2.

#### Damage source location between the intact and restored sandstone

**Fig 6:**
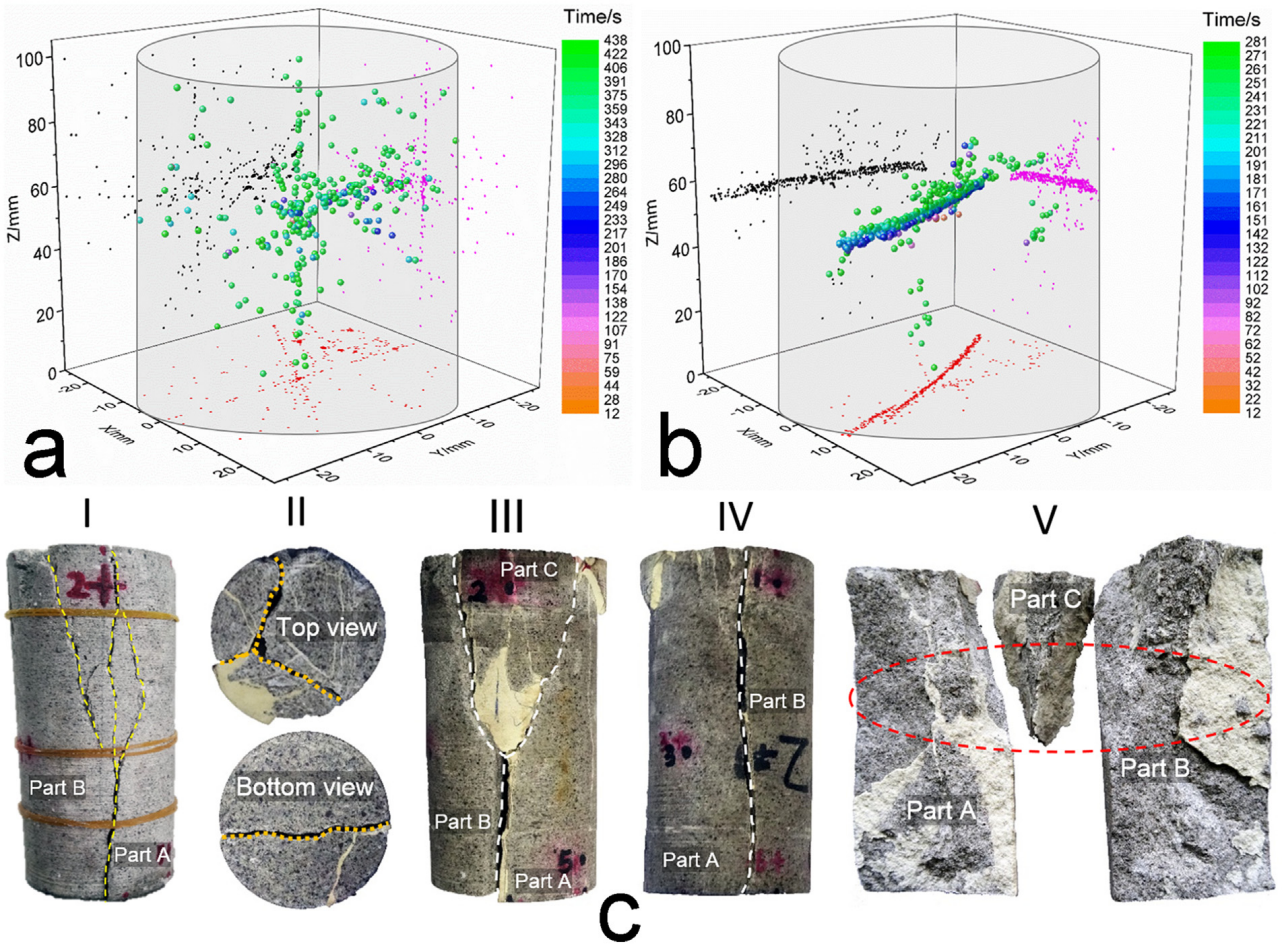
Damage source locations that were captured by an AE test. **(a)** Damage source distribution for the intact sandstone No. 1. **(b)** Damage source distribution for the restored sandstone No. 1. **(c)** Fracture modes of the intact sandstone and fractured sandstone (No. 1): I, Front view for the fracture mode of intact sandstone; II, Fracture mode for the ends, where the golden dashed line indicates the fracture form of the restored sandstone; III and IV, Front views for the fracture mode of the restored sandstone; V, Stripped parts from the fractured restored sandstone, where the red ellipse indicates the damage source concentration zone. The surface color changes between the intact sandstone and the restored sandstone are attributed mainly to the Vaseline painting to facilitate demolding during the restoration process.

To locate the AE source in the specimens, we utilized six sensors that were mounted around the surface of the specimen to determine the three-dimensional locations of the AE events. In terms of the acquired data, the AE source location patterns for the intact and restored sandstones are plotted in Fig 6. The phenomenon of bare AE activity around the end areas during the initial loading process demonstrates the effectiveness of the coated mixture of Vaseline and stearic acid at the ends of the specimen.

The most intuitional difference between the different options is that the intact sandstone shows an overall dispersed distribution of AE events, whereas the distribution for the AE events in the restored sandstone is relatively centralized to a certain area. As an interpretation for the AE events’ distribution in the intact sandstone under uniaxial compression, specific location characteristics can be seen in Table 3, and the following characteristics can be summarized in terms of Table 2: (1) few AE events occur before the crack closure threshold (0–225.27 s), and their locations are positioned around the center axis of the specimen, where the range for the Z coordinates is 40–60 mm, which indicates the initial damage source in the intact sandstone; (2) as the load increases, several sporadic events are captured between the crack closure threshold and the crack initiation threshold (225.27–340.62 s), but the distribution pattern still shows some similarities with what was illustrated in stage (1), except for the magnitude increase in AE activity; and (3) this stage ranges from 340.62 s to the final fracture of the specimen; the AE activity during this period becomes more intense and the events rapidly increase; the crack starts to propagate to the full scope of the rock mass, and this process is accompanied by crack propagation, crack damage, and crack coalescence until some macroscopic cracks appear. Based on the three-dimensional damage source locations and the locations in the respective XZ planes and YZ planes, the crack evolution process starts from somewhere in the center of the rock mass before the cracks spread to the upper and lower parts of the rock mass. Moreover, intersecting lines can be observed based on the locations in the XY plane, and a vertical line can be observed based on the vertical line in the XZ place, which could provide insights regarding the final fracture mode.

**Table 3:** Specific parameters for the damage source locations based on AE detection.

Specimen	0-TC	TC-TI	TI-TD	TD-TP	Total events
Intact sandstone					
Time range (s)	0–225.27	225.27–340.62	340.62–436.84	436.84–436.84	
Events	133	214	494	0	841
Restored sandstone					
Time range (s)	0–155.84	155.84–213.32	213.32–244.79	244.79–259.83	
Events	1490	303	148	61	2002

Fig 6(c), which is presented as an auxiliary argument for the fracture form of intact sandstone, shows the actual fracture modes of the intact sandstone and restored sandstone. The actual fracture modes indicate that the initial intact sandstone approximately split into two symmetric parts (parts A and B) that were based on the shapes in sections III, IV, V, and the bottom view in section II (these modes do show the fracture forms of the restored sandstone, but the main fracture traces under the secondary compression generally followed those of the first compression), and the inner section view shows a general flat fracture face; these two appearances coincide with the vertical line in the XZ plane. The top view of section II shows a three-crack intersection pattern, which is in accordance with the intersecting lines on the XY plane.

The distribution of AE events for the restored sandstone shows a spatial ribbon-like aggregation, and the majority of damage sources occur at approximately 55 mm along the Z axis direction. In addition, an orthographic projection on the XY plane and the sides’ projection on the XZ and YZ planes also exhibit ribbon-like distributions. Most of the damage sources are horizontally located in the middle area of the specimen based on the sides’ projections. According to Table 2, these locations can be divided into three groups by the same measure that was used in the analysis on the intact specimen. The first group includes the locations that occur between 0 and 155.84 s, and a large amount of damage has been detected in this period. Acute damage occurred, and corresponding AE emissions were frequently captured. However, the evolutions of the inner cracks were extremely drastic; they propagated and coalesced with one another and formed inner macroscopic cracks. The damage sources concentrated together and overlaid with one another, which created an embryonic form with a ribbon-like distribution. The second group is divided by the period that ranges from 155.84 to 213.32 s, and the AE events were comparatively smaller than those in the first group, but the detected events still gathered together and formed a general pattern with a ribbon-like distribution. The third and fourth groups range from 213.32 to 244.79 s and from 244.79 s to the final fracture, respectively. The AE activity in the third group was similar to the activity that occurred in the second group, and the damage sources still gathered together around the ribbon-like area. During the later period, some locations began to shift to the upper and lower directions of the ribbon-like area. This shift indicates the occurrence of large macroscopic cracks, which determine the upcoming moment of the peak stress. What occurs in the last group can be summarized by the AE activity dispersion, which directly led to the final failure of the restored sandstone. The detailed parameters are listed in Table 3, which shows that the total events of the restored sandstone were considerably larger than those of the intact sandstone, which reflects the strong propagation and coalescence of the cracks in the restored sandstone. Grouting measures could be effective to some extent, but they cannot improve the cracks’ evolution during the pre-peak procedure.

The actual fracture modes of the restored sandstone (Fig 6(c)) demonstrate that the fracture trace lines/surfaces were nearly identical to those of the intact sandstone, and the direct cause for the final failure was twofold. The first cause occurred during the earlier stage of compression, which was the crack propagation along the flake-like adhesive that was composed of MEYCO 364; the position for the propagation trace line coincides with the position of the ribbon-like area in Fig 6(b). The second cause, which occurred during the later stage, was interface decoupling between the surface of the rock mass and the adhesive, which initiated from the ribbon-like area and spread to the two ends of the specimen; this phenomenon confirms the AE locations that are described for the third group in the prior paragraph.

Additionally, the inner fracture mode can reveal the mechanism behind the plummeting axial strains in *σ*_cc_ and *σ*_p_ before and after restoration in Figs 4(a) and 5(a). Most inner macroscopic cracks were filled by MEYCO 364, so the restored sandstone underwent a comparatively quiet period during its initial AE activity (see Fig 5(b)). Compared to this scenario, the intact sandstone had natural imperfections in its inner microscopic cracks, which directly caused a relatively larger axial strain than the restored sandstone when *σ*_cc_ was reached. Moreover, the inner fracture indicates that interface slipping caused the final failure, and the rock-adhesive interface could not sustain slippage that was comparatively larger than the intact rock mass could sustain, which explains the decrease in the axial strain before and after restoration when *σ*_p_ was reached. Additionally, the time that was needed to reach *σ*_p_ for the restored sandstone was considerably less than that required by the intact sandstone under an identical loading rate.

### Test results for coal specimens

The test results of coal specimens have a number of unique features compared with sandstone specimens because of differences in their mechanical properties, and the strength of cracked coal could be substantially improved by MEYCO 364.

#### Test on intact coal

**Fig 7:**
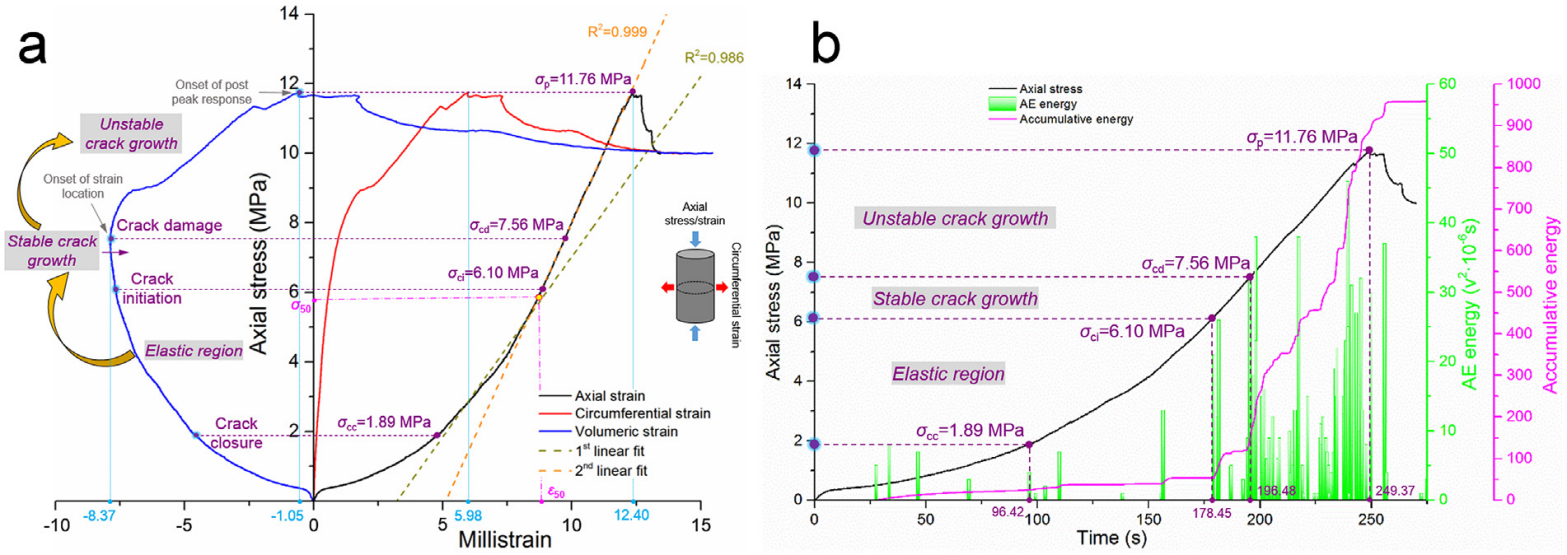
Test results of intact coal No. 2. **(a)** Relationship between the axial/circumferential/volumetric strain and axial stress under uniaxial compression. **(b)** Relationship between the time and axial stress/AE energy/accumulative AE energy under uniaxial compression.

Two coal specimens were tested together, and the results of coal No. 2 are presented in this section. Fig 7 shows the mechanical properties and AE energy details of the intact coal No. 2. In terms of the identical analytical measures from the ‘Test results for sandstone specimens’ section, the crack damage threshold (*σ*_cd_) can be easily determined by the reversal sign from the negative extension to positive extension in the volumetric strain-stress curve in Fig 7(a); this threshold, which represents the onset point of the strain location, is 7.56 MPa. The crack closure threshold (*σ*_cc_) is determined based on the turning point from the first concave section to the linear section along the stress-axial strain curve in Fig 7(a) and multiple curves in Fig 7(b), and the value for *σ*_cc_ is 1.89 MPa. The crack initiation threshold (*σ*_ci_) is defined when the axial strain-stress curve turns into a sharp increasing trend, and no further long-term intermediate plateau occurs along the time-accumulative energy curve; thus, the value for *σ*_ci_ is defined as 6.10 MPa. Correlated parameters are listed in Table 4. The maximum axial strain is considerably smaller than that of sandstone under an identical loading rate (which is controlled by the displacement mode), and the time that was needed for each stage is also smaller (based on a comparison between Tables 2 and 4). These phenomena indicate that the initial inherent crack occurrences were extremely prosperous, and the sealed cracks were abundantly activated even under relatively small axial strain; the microscopic cracks propagated into macroscopic cracks in a short period of time, and the fracture procedure was more acute than that of the sandstone. These diversities are reasonable in view of the macrostructural interpretation between coal and sandstone. Different support schemes that are inspired by these differences should be applied because of the different fragmentary degrees of coal roadways and rock roadways in our field practices.

**Table 4:** Key test parameters for the intact coal and restored coal (No. 2).

Specimens	*σ*_cc_ (MPa)	*σ*_ci_ (MPa)	*σ*_cd_ (MPa)	*σ*_p_ (MPa)	*E* (GPa)	*E*_50_ (GPa)	MV* (E-3)	MC* (E-3)
Intact coal	1.89 (0.16)	6.10 (0.52)	7.56 (0.64)	11.76	1.34	0.67	-8.37	5.98
Restored coal	2.06 (0.15)	5.93 (0.42)	7.07 (0.50)	14.15	1.14	0.63	-10.23	14.12
Specimen	MA* (E-3)	TC* (s)	TI* (s)	TD* (s)	TP* (s)	TC-TI* (s)	TI-TD* (s)	TD-TP* (s)
Intact coal	12.40	96.42	178.45	196.48	249.37	82.03	18.03	52.89
Restored coal	16.19	157.71	239.09	253.94	350.76	81.38	14.85	96.82

^#^ Value in the bracket is defined as the ratio between the axial stress of each threshold and the peak stress.

* MV, MC, and MA represent the maximum absolute volumetric/circumferential/axial strain before fracturing, respectively. TC, TI, TD, and TP are the moments when *σ*_cc_, *σ*_ci_, *σ*_cd_, and *σ*_p_ are reached, respectively, and TC-TI, TI-TD, and TD-TP are the durations between TC and TI, TI and TD, and TD and TP, respectively.

#### Tests on restored coal

Coal specimens that have undergone uniaxial compression showed a high degree of breakage, but they retrieved their integrity after the grouting procedure with MEYCO 364; the operation process was identical to the process that was conducted on the sandstone.

**Fig 8:**
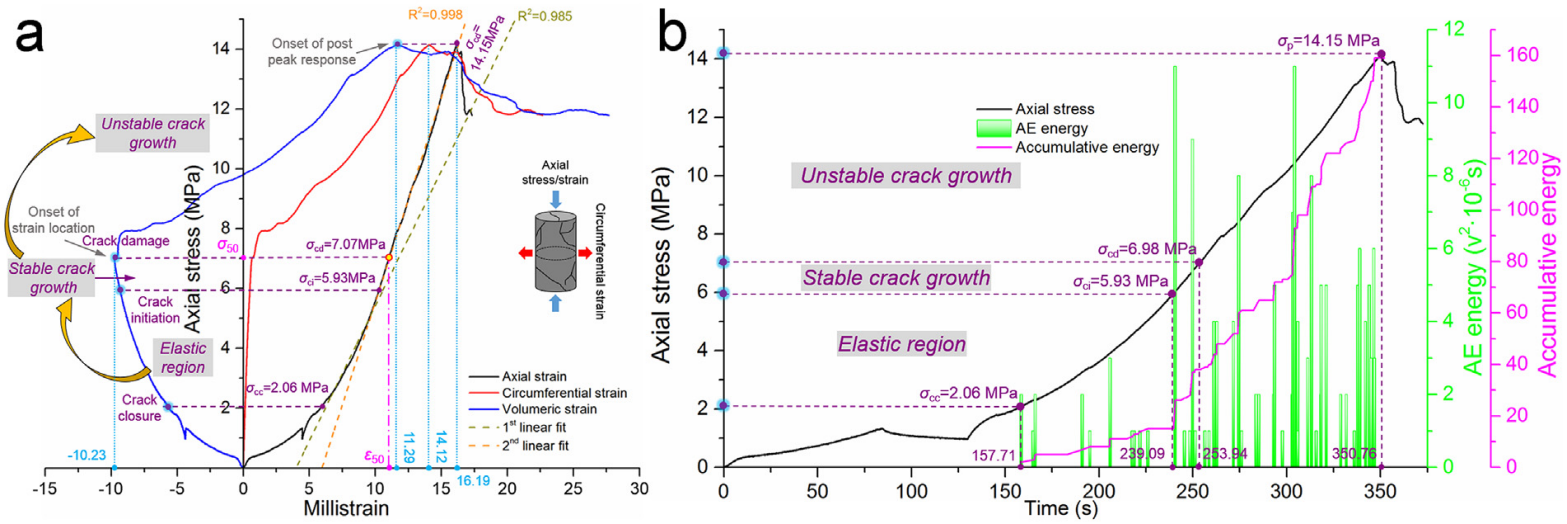
Test results of restored coal No. 2. **(a)** Relationship between the axial/circumferential/volumetric strain and axial stress under uniaxial compression. **(b)** Relationship between the time and axial stress/AE energy/accumulative AE energy under uniaxial compression.

Fig 8 presents the test results of the restored coal No. 2. The peak stress of the restored coal shows an even higher value than the value of its initial intact coal; the value is 14.15 MPa, which has increased by 20.3% based on the initial peak stress of 11.76 MPa. Furthermore, the strength recovery coefficient increased to 1.20 even though the elastic modulus was slightly decreased. The strength restoration with the grouting measure is preferred from the perspective of practical field applicability and the reliability of the obtained improvements. In other respects, the earlier stage of acoustic energy emission is quite rare, which shows a high consistency with what the restored sandstone No. 1 expressed during its earlier stage. These findings demonstrate that damage in a specimen that is restored by grouting cannot be detected during its earlier fracture stage; furthermore, microscopic cracks closed in these specimens under compression.

The crack damage threshold (*σ*_cd_) is defined in view of the reversal sign along the volumetric strain-stress curve and is marked by the onset of the strain location. This point also separates the stable crack growth procedure and unstable crack growth. In the same way as in the aforementioned sections, the crack closure threshold (*σ*_cc_) and crack initiation threshold (*σ*_ci_) are easily marked in Fig 8. All the key parameters that are associated with Fig 8 are listed in Table 4.

All the threshold coefficients (the ratio between a threshold and peak stress) of the restored coal are lower than those for the initial intact coal, which indicates that the crack propagation in the restored coal occurred earlier and faster than for the intact coal. In addition, all the values of the maximum absolute volumetric strain, maximum absolute circumferential strain, and maximum absolute axial strain in the restored coal were larger than those for the intact coal. MEYCO 364 can improve the peak stress of a coal mass to a certain extent, and its use is particularly effective for the soft and inherent cracks in developed rock masses. Nevertheless, unbonded and slightly bonded cracks in the coal are considerably easier to propagate and coalesce with others; hence, macroscopic cracks are more inclined to emerge, and the absolute strain values increase. Table 4 presents a direct demonstration of this phenomenon. In field practice, an overly large deformation of an area of grouted coal mass could occur, but such an occurrence does not imply a total failure of its bearing capacity.

#### Damage source location between intact and restored coal

**Fig 9:**
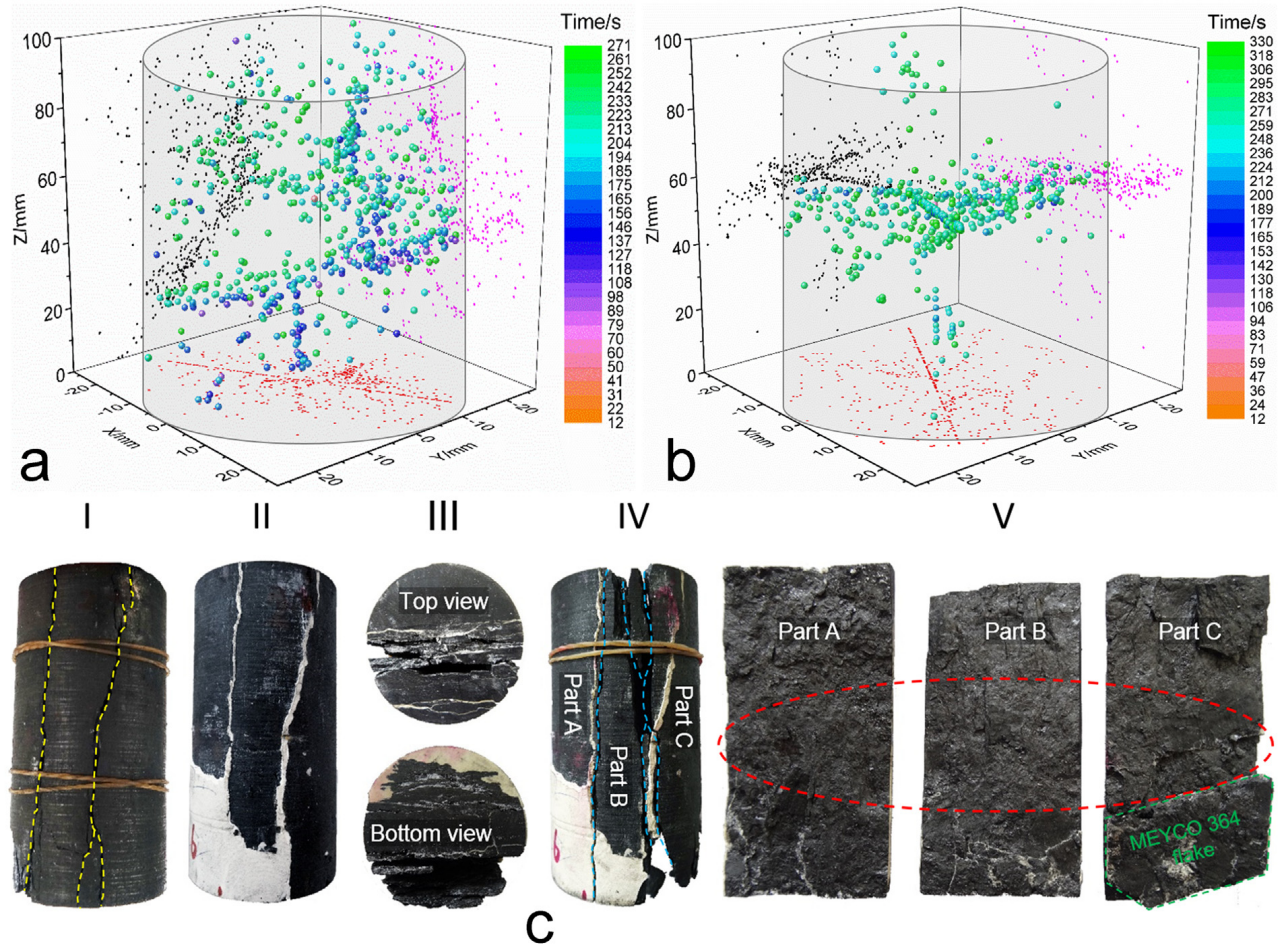
Locations of events that were captured by an AE test. **(a)** Coal No. 2 during the first round test, with a total of 751 events, where 54 events occurred during 0–140 s, 473 events during 140–250 s, and 224 events during 250–314 s. **(b)** Coal No. 2 during the second round test, with a total of 487 events, where 4 events occurred during 0–130 s, 83 events during 130–240 s, and 400 events during 240–330 s. **(c)** Fracture modes of the intact coal and restored coal. I, Fracture mode of the intact coal. II, Front view of the restored coal. III, End view for the fracture modes of the restored coal. IV, Front view for the fracture mode of the restored coal. V, Stripped parts from the fractured restored coal, where the red ellipse indicates the damage source concentration zone.

The damage source locations for the intact coal and restored coal are plotted in Fig 9, and some final fracture modes are also included. A comparative study indicates that the location points of restored coal are lesser than the location points of intact coal, which are inspired by the damage source location details of sandstone, indicating that the strength improvement by MEYCO 364 could lead to a decline in the damage source locations. The relative parameters are listed in Table 5.

**Table 5:** Specific parameters for damage source locations based on AE detection.

Specimen	0-TC	TC-TI	TI-TD	TD-TP	Total events
Intact coal					
Time range (s)	0–96.42	96.42–178.45	178.45–196.48	196.48–249.37	
Events	9	163	87	328	587
Restored coal					
Time range (s)	0–157.71	157.71–239.09	239.09–253.94	253.94–350.76	
Events	2	83	56	345	486

Damage detection in intact sandstone exhibits a dispersed distribution. Only slight damage was detected during the initial point to the crack closure point, and then a rush of scattered damage sources occurred during the elastic stage of the compression procedure. Finally, damage sources were increasingly detected during the stable crack growth stage and unstable crack growth stage, and they migrated to the end areas in the coal specimen, which indicates a thorough fracture of the coal. The following key points are reached based on side projections on the XY and YZ faces: (1) an obvious inclined contour line occurs on the XY face, which conforms to the end fracture forms in the two ends, where several split-cracks are tracked on the end surfaces. The eventual fracture shape is integrally shown in the leftmost photo (I) in Fig 9(c) and traceably shown in the third photo (III) in Fig 9(c); (2) the lower-middle part damage source locations on the YZ face show an inclined ribbon-like distribution, and the locations on the upper part are quite scattered, which indicates some constituent of shear fracture during the loading process.

Damage source detection on the restored coal shows a comparatively concentrated distribution, and damage barely occurred during the crack closure procedure; hence, the increase in the volumetric/axial/circumferential strain was mainly caused by crack closure or slight propagation, which embodies the effectiveness of the adhesive material. Then, the inner AE activity began to increasingly activate during the elastic stage, stable crack growth stage, and unstable crack growth stage. Most of the locations were detected in the unstable stage, which demonstrates that the adhesive material observably altered the mechanical properties of the coal; pre-existing cracks or excavation-induced cracks effectively avoided being propagated with neighboring ones, which is the eminent reason why the strength of the coal was upgraded. Most of the damage sources were located around the central area in the coal and then spread to the end directions because of the projections on the XZ and YZ faces; the contour line along the XY face indicates a split-induced fracture, which is consistent with the implications of Fig 9(c).

The fracture modes in Fig 9(c) are different from those of the sandstone; the fractured traces that were caused by secondary compression on the restored sandstone nearly followed those from the first compression on the intact sandstone, and the fracture was dominated by interfacial decoupling between the sandstone and MEYCO 364. Nevertheless, the stripped failure parts of Fig 9(c) demonstrate that the stick-slip movement that caused the final macroscopic failure of the coal was mainly composed of two aspects. The primary aspect was slippage-induced failure in the adhesive coal, and the secondary aspect, which shares a certain similarity with what occurred on the restored sandstone, was interfacial decoupling between the coal and MEYCO 364. Hence, another factor increased the strength of the coal. The reason could be linked with the porosity of the coal [39].

### Test results for MEYCO 364 specimens

**Fig 10:**
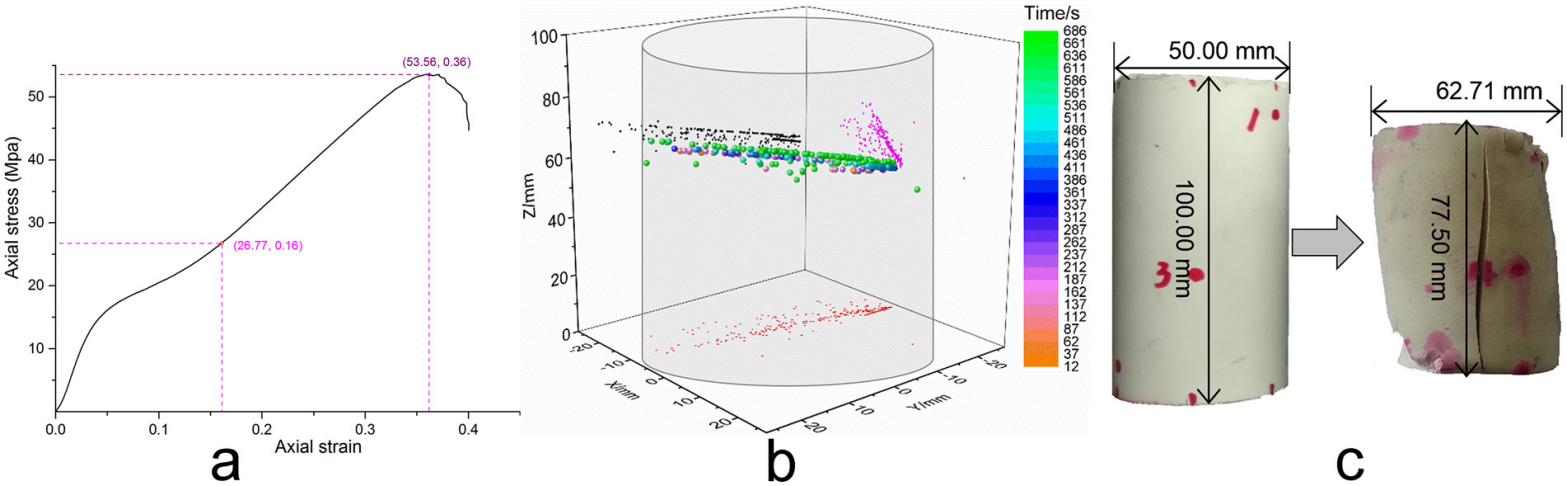
Test results of MEYCO 364. **(a)** Relationship between the axial strain and axial stress. **(b)** Damage source location based on AE detection. **(c)** Scale change before and after the uniaxial compression test.

With the expectation of acknowledging the mechanical properties of MEYCO 364, we prepared a standard specimen made from this material; the diameter and height were 50 and 100 mm, respectively. Because of the contradiction between the overly large circumferential strain and limited chain length, we failed to measure the final circumferential strain and volumetric strain; the circumferential extensometer was avalanched from the chain. We had not expected this contraction before the test on the MEYCO 364 specimen. The deformed specimen is shown in Fig 10(c), and the scale change rates for the diameter and height are 1.25 and 0.78, respectively. The circumstantial strain was too large for a normal extensometer to measure, and the deformation reflects its elastic property.

Fig 10 shows the strain-stress curve, damage source location, and scale change of the specimen. The strain-stress curve indicates that a rapid linear increase occurred first, followed by a slow concave-shaped increase. This form change is a notable difference from a typical test on brittle materials, such as glass and rock. The MEYCO 364 specimen was a man-made chemical adhesive, and the specimen was homogeneously mixed by resin and catalysts; this procedure avoids the generation of inner cracks. Hence, the rapid increase was mainly caused by its nonporous inner structure, and crack closures did not appear. The turning point indicates an emergency in the inner cracks, and the concave section after the turning point was induced by the closure of those cracks. The damage source location in Fig 10(b) shows accurate positions for these cracks, which coalesced together around the center of the specimen and then propagated to the surface to create a final macroscopic failure, as shown in Fig 10(c).

The aforementioned analysis demonstrates that the chemical high polymer adhesive MEYCO 364 is remarkable in reinforcing fractured rock mass because of its high strength under large deformations, which represents an advantage compared to superfine cement or Portland cement.

## Conclusions

The following conclusions can be drawn based on the results of this study:

1. End-coating with a mixture of Vaseline and stearic acid is an effective and convenient measure to eliminate the end effects during general uniaxial compression on a rock specimen. It can reduce the noise that is caused by fretting between the compression plate and the end of the specimen during the AE signal acquisition.
2. The strength recovery coefficient of a cracked specimen is less than 1 if its initial intact peak stress is larger than that of the adhesive (MEYCO 364 in this study). Hence, the restoration capability of MEYCO 364 on sandstone is limited under this circumstance; the cracked trace lines/faces of the restored sandstone nearly follow the cracked trace lines/faces of the intact sandstone under uniaxial compression, and interfacial decoupling between the rock mass and adhesive is the primary inducement to the lead failure of restored sandstone. Nonetheless, grouting reinforcement can still provide certain effects for this type of rock mass and has some referential significance to engineering practices.
3. The strength restoration ability of MEYCO 364 on coal is remarkable, and a recovery coefficient that surpasses 1 is normal because of the comparatively larger peak stress of MEYCO 364. The failure of restored coal is mainly caused by interfacial decoupling between coal and MEYCO 364 and self-fractures in the coal because of crack propagation/coalescence. Additionally, restored coal can sustain a relatively larger deformation and maintain its bearing capacity at the same time.
4. Overall, the time-AE energy curve and time-accumulative energy curve prove that both the restored coal and the sandstone undergo a quiet duration before the crack closure threshold (*σ*_i_), whereas the AE activity of the intact coal and the sandstone start behaving as the compression actuates. The three-dimensional damage source locations indicate that AE events are closely associated with the strength recovery coefficient, and the number of AE events of a restored rock mass shows an increasing tendency if the coefficient is less than 1, whereas the number of a restored rock mass shows a decreasing tendency if the coefficient is larger than 1. In addition, the location details are in good agreement with the actual fracture modes of the specimens.
5. The chemical adhesive MEYCO 364 shows superiorities in reinforcing fractured rock masses, which can be popularized in field practices; the advantages are particularly apparent for rock masses whose initial peak stress is lower than that of the chemical material, such as coal or soft rock. Because of its highly plastic nature, MEYCO 364 can sustain a high bearing capacity even under large deformation conditions. Hence, grouting reinforcement that is aided by MEYCO 364 or a similar chemical adhesive should be preferentially adopted compared to some other competing products, such as superfine cement or sulfate aluminum cement.

## Supporting Information

S1 File Circumferential extensometer strain calculations. (DOCX)
