# Supplementary material for: Strength Restoration of Cracked Sandstone and Coal under a Uniaxial Compression Test and Correlated Damage Source Location Based on Acoustic Emissions
Source: PLoS One. 2015 Dec 29;10(12):e0145757. doi: 10.1371/journal.pone.0145757 (PMC4694704; doi:10.1371/journal.pone.0145757)
Supplement: S1 File — (DOCX) [file pone.0145757.s001.docx]

**S1 File. Circumferential extensometer strain calculations.** (DOCX)

A specimen under uniaxial compression will radially deform, and the circumferential extensometer measures the change in the chord length (*l_f_*-*l_i_*) between the center of the two end rollers in the chain and not the change in the specimen’s circumference, as shown in the figure below. This section describes the calculations that are used to compensate for this difference.

**S1 Fig. 1 Schematic diagram of the deformation pattern of the circumferential extensometer (end roller)**. Where *l_i_*, *l_f_*, *r*, *R_i_*, and *R_f_* represent the initial cord length, final cord length, radius of roller, initial radius of the specimen, and final radius of the specimen, respectively. Here, *θ_i_*, and *θ_f_* represent the angle that is subtended by the initial chord length and final chord length, respectively, in radians.

The chain length remains constant during the entire testing procedure:

$l_{c}=(R_{i}+r)(2\pi-\theta_{i})=(R_{f}+r)(2\pi-\theta_{f})$ (S1)

$l_{c}=(R_{i}+r)(2\pi-\theta_{i})=(R_{i}+\Delta R+r)\left[2\pi-(\theta_{\text{i}}+\Delta\theta)\right]$ (S2)

where ∆*R* is the change in the specimen’s radius and *∆R*=*R_f_*-*R_i_*. ∆*θ* is the change in the angle that is subtended by the chord length, in radians, where *∆θ*=*θ_f_*-*θ_i_*.

Based on Eqs. (S1) and (S2), the following equation can be obtained:

$2\pi R-\Delta R\theta_{\text{i}}\text{=}R_{\text{i}}\Delta\theta+\Delta R\Delta\theta+r\Delta\theta$ (S3)

Assuming that ∆*R*∆*θ*≪1 (i.e., ≈0), Eq. (S3) can be written as follows:

$\Delta R(2\pi-\theta_{i})\text{=}\Delta\theta(R_{i}+r)$ (S4)

The equations for *l_i_* and *l_f_* are listed below:

$l_{i}=2(R_{i}+r)\sin(\frac{\theta_{i}}{2})$ (S5)

$l_{f}=2(R_{i}+r)\sin(\frac{\theta_{i}}{2})+\Delta l\text{=}2(R_{i}+\Delta R+r)\sin(\frac{\theta_{i}+\Delta\theta}{2})$ (S6)

Because of the small angles (∆*θ*≪1),

$\sin(\frac{\theta_{i}}{2}+\frac{\Delta\theta}{2})=\sin(\frac{\theta_{i}}{2})\cos(\frac{\Delta\theta}{2})+\cos(\frac{\theta_{i}}{2})\sin(\frac{\Delta\theta}{2})\text{， at the same time, }\cos(\frac{\Delta\theta}{2})\approx1\text{ and }\sin(\frac{\Delta\theta}{2})\approx\frac{\Delta\theta}{2}$ (S7)

By combining Eqs. (S6) and (S7) and because ∆*R*∆*θ*≈0, the following equation can be obtained:

$\frac{\Delta l}{2}=\Delta R\sin(\frac{\theta_{i}}{2})+\frac{\Delta\theta}{2}\left(R_{i}+r\right)\cos\left(\frac{\theta_{i}}{2}\right)$ (S8)

where ∆*l* is the extensometer output and ∆*l* =*l_f_*-*l_i_*.

By combining Eqs. (S4) and (S8), ∆*R* can be expressed as

$\Delta R\text{=}\frac{\Delta l}{2\left[\sin(\frac{\theta_{i}}{2})+(\pi-\frac{\theta_{i}}{2})\cos(\frac{\theta_{i}}{2})\right]}$ (S9)

because

$\Delta R\text{=}\frac{\Delta C}{2\pi}$ (S10)

where ∆*C* is the change in the specimen’s circumference and ∆*C*=*C_f_*-*C_i_*. *C_f_* (2*πR_f_*) and *C_i_* (2*πR_i_*) are the initial circumference and final circumference of the specimen, respectively.

Finally, based on Eqs. (S9) and (S10), the change in the specimen’s circumference (∆*C*) is expressed as

$\Delta C\text{=}\frac{\Delta l\pi}{\sin(\frac{\theta_{i}}{2})+(\pi-\frac{\theta_{i}}{2})\cos(\frac{\theta_{i}}{2})}$ (S11)

Because the length of the chain around the specimen does not change, we obtain

$\theta_{i}=2\pi-\frac{l_{c}}{(R_{f}+r)}$ (S12)

Consequently, the measured change in the chord length (∆*l*) can be transformed into the change in the specimen’s circumference (∆*C*) by Eqs. (S11) and (S12), which is well within the accepted limits of the required accuracy. Hence, the circumferential strain can be calculated as follows:

$\varepsilon_{\text{c}}=\frac{\Delta R}{R_{i}}=\frac{\Delta C}{C_{i}}$ (S13)
